# A lesson for post-COVID healthcare: assessment of physical and psychosocial risk factors on perceived pain intensity among urban individuals

**DOI:** 10.3389/fpsyg.2024.1447168

**Published:** 2025-01-16

**Authors:** Hung Chak Ho, Wentao Bai, Stanley Sau-Ching Wong, Chi Wai Cheung

**Affiliations:** ^1^Department of Public and International Affairs, City University of Hong Kong, Kowloon, Hong Kong SAR, China; ^2^Social Determinants of Health Initiative, City University of Hong Kong, Kowloon, Hong Kong SAR, China; ^3^Department of Anaesthesiology, School of Clinical Medicine, The University of Hong Kong, Pok Fu Lam, Hong Kong SAR, China; ^4^Hong Kong Sanatorium & Hospital, Happy Valley, Hong Kong SAR, China

**Keywords:** perceived pain intensity, psychosocial factors, personal wellbeing, mental distress, insomnia, risk factor

## Abstract

**Background:**

Perceived pain intensity is an important determinant of health-related quality of life. A lack of studies has investigated the co-influences of physical and psychosocial risk factors on perceived pain intensity and the shifts in effects after pandemic. As a post-COVID symptom, it is important to re-assess the risk factors for post-COVID heath care.

**Methods:**

Four dimensions of physical/psychosocial risk factors were assessed: medical history, personal wellbeing and psychological distress, lifestyle, and socio-demographic characteristics. We first identified subgroups with significant increase in perceived pain intensity after pandemic by a comparison with the baseline group (all participants). Based on the variables associated with a severe increase in pain score (NRS), multivariate regression models were applied to identify risk factors on perceived pain intensity.

**Results:**

Among 3,237 urban individuals in Hong Kong, 20.95 and 30.58% were with severe pain (NRS > = 4) before and after pandemic. Participants with respiratory disease had the most significant increase in perceived pain intensity (increase in NRS: 1.29 [0.65, 1.93]), seconded by those with known psychiatric diseases and living with special needs. After pandemic, insomnia, known psychiatric diseases, female, and low household income remained as significant risk factors, and insomnia remained as the most significant (estimate: 1.018 [CI: 0.814, 1.221]). The effect sizes of these factors were increased after pandemic. Respiratory disease, cardiovascular disease, and low education (secondary school or below) were additional risk factors.

**Conclusion:**

It is necessary to develop up-to-date interventions targeting vulnerable populations, particularly individuals with known psychiatric diseases and insomnia, for pain reduction.

## Introduction

Pain is a leading cause of health burdens worldwide (Kuehn, 2018). Particularly, perceived pain intensity is an important determinant of health-related quality of life (Obradovic et al., 2013). For example, EQ-5d is one of the known instruments to measure health-related quality of life, and perceived pain intensity is one of the five major dimensions of EQ-5d (Obradovic et al., 2013). Furthermore, pain is not only causing health burden but also leading to a huge economic loss. One example is that around 20% U.S. individuals suffered from chronic pain, costing the country $560 billion in medical costs each year (Kuehn, 2018). Thus, identifying risk factors causing perceived pain intensity is necessary in order to target vulnerable subpopulations for preventive medicine and intervention, based on the prioritization of appropriate treatments and action plans to mitigate the pain consequences among high-risk groups.

Based on the biopsychosocial model of pain (Turk et al., 2011), perceived pain intensity is not only a physiological health outcome but also a psychological outcome that affect wellbeing and quality of life. As such, previous studies have found that perceive pain intensity is not only affected by individual-level physical risk factors but also psychosocial risk factors. Regarding physical and psychosocial risk factors of pain, it can be categorized by the following dimensions: (1) medical history, (2) personal wellbeing and psychological distress, (3) lifestyle, and (4) socio-demographic characteristics.

Among all factors related to medical history, (1) respiratory diseases, (2) cardiovascular diseases, (3) cancer, (4) known psychiatric diseases, and (5) insomnia are common determinants that can induce comorbidity issues to affect pain intensity (Ha et al., 2014; Karimi et al., 2023; Mangolianshahrbabaki et al., 2024; Vardar-Yagli et al., 2024; Serpas et al., 2024). For example, a study in Korea investigated 13,841 individuals aged 20–89 and discovered that those with a history of cardiovascular disease were more likely to have chronic low back pain (Ha et al., 2014). A meta-analysis for 49 articles among 120,489 individuals found a moderation effect of sleep quality on the relationship between pain and depression. Serpas et al. (2024) also found a serial mediation effect between sleep quality, depressive symptoms, and pain. Regarding personal wellbeing and psychological distress, subjective wellbeing and self-rated mental health can both affect pain issues (Blyth et al., 2001; Raftery et al., 2011; Chen et al., 2012; Amo-Agyei and Maurer, 2024). For example, an early study in Australia found that individuals with chronic pain frequently experienced opioid-related problems, psychological distress, and a poor quality of life (Blyth et al., 2001). This study examined 17,543 individuals and found that older age, female, low education level, without private health insurance, unemployed for health reasons, poor self-rated health, and high psychological distress could be risk factors for chronic pain in Australia. Similar results have been found in Ireland (Raftery et al., 2011) and China (Chen et al., 2012). Based on a study of 21,783 respondents using a subjective wellbeing index developed by World Health Organization (WHO), personal wellbeing is lower among individuals with pain (Amo-Agyei and Maurer, 2024). Regarding lifestyle factors, smoking status and alcohol consumption are significantly associated with pain (Smuck et al., 2020; Ditre et al., 2023). For example, a study with 2,307 individuals from the National Health and Nutrition Examination Survey found that smoking can be associated with pain in almost all body regions (Smuck et al., 2020). Ditre et al. (2023) found that alcohol consumption may be a casual factor of pain. Furthermore, a study in Netherland investigated physical, lifestyle, psychological and social determinants of pain among depressed older adults and found that several biopsychosocial determinants (especially anxiety) were associated with both acute and chronic pain intensity (Hanssen et al., 2014). The above studies indicated that perceived pain intensity can be affected by multiple risk factors.

Despite the fact that a wide range of risk factors have found, it is still uncertain how these risk factors can co-influence perceived pain intensity, especially among urban individuals. It is important to note that pain issues of urban individuals are often affected by lifestyle and daily behaviors, such as daily work (Cheung et al., 2017). Specifically, urban lifestyle and daily behaviors may not only affect physiological responses but also inducing psychological distress among local individuals, which further increase the negative impacts on perceived pain intensity. More importantly, it is also unknown how physical and psychosocial risk factors of pain among urban individuals have been shifted after the pandemic. As pain is a one of the major long COVID symptoms, it is necessary to comprehensive assess the co-effect of risk factors among urban individuals in order to enhance post-COVID healthcare.

Thus, this study assessed the co-effects of physical and psychosocial risk factors on perceived pain intensity. A population-based study was conducted in Hong Kong because urban individuals in Hong Kong are often suffered from pain issues due to daily lifestyle and compact living environment. Three questions were posed: (1) Which subgroups of urban individuals had higher perceived pain intensity when compared to the general population? (2) Which subgroups had individuals with a greater magnitude of increased perceived pain intensity over time? (3) Did the risk factors for perceived pain intensity shift after the pandemic? The findings were aimed to identify shifts in vulnerable subgroups for pain management and health action programs.

## Data and methods

### Population-based survey

The data were retrieved from the 3rd collection of the UrbanAB-C (Urban Accessibility and Behaviors–cohort). UrbanAB-C was a population-based cohort based on multiple cross-sectional online surveys to assess urban wellbeing, neighborhood accessibilities and barriers, socio-psychological responses, and disaster behaviors before and after COVID-19 in Hong Kong. The third wave was conducted between October 18 and October 24, 2022.

To facilitate the participant’s recruitment during the critical moment of pandemic, UrbanAB-C data were collected through an online platform with a public participation geographic information system (PPGIS) through the PopPanel operated by Hong Kong Public Opinion Research Institute (HKPORI). PopPanel was a joint panel of (1) a probability-based recruitment randomly through telephone surveys and representative of the Hong Kong population, and (2) a non-probability-based recruitment from volunteers through online registration (Sit et al., 2021), which all panel members were continuously invited to participate in different surveys. PPGIS was an online platform to allow participants to provide volunteered geographic information (VGI) including location matters through maps and other tools.

Through PopPanel, invitations of the 3rd data collection were sent via email. A total of 83,339 Hong Kong individuals aged > = 15 were invited. However, due to the possibility of being identified as spam/junk mails, there were only 4,702 panel members clicked on the link of invitation and started the survey. About 68.9% of respondents (*n* = 3,237) have completed the questionnaire.

### Health outcomes

Perceived pain intensity was measured based on the 11-point numerical rating scale (0–10) of pain level (Frampton and Hughes-Webb, 2011). This numeric rating scale (NRS) indicated no pain as “0” and the worst pain imaginable as “10,” and has been widely validated for measuring perceived pain intensity. Previous studies have commonly defined NRS > =4 as the optimal cut-off of people with moderate and severe pain and in need of pain treatment, versus those were only with mild or no pain (score < =3) (Lahtinen et al., 2006; Ledowski et al., 2006; Gerbershagen et al., 2011).

We categorized physical and psychosocial risk factors into four dimensions: (1) medical history, (2) personal wellbeing and psychological distress, (3) lifestyle, and (4) socio-demographic characteristics.

Factors related to medical history were measured based on self-reported comorbidity information: (1) respiratory diseases, (2) cardiovascular diseases, (3) cancer, (4) known psychiatric diseases, and (5) insomnia. Factors of personal wellbeing and psychological distress were measured by (1) self-rated mental health and (2) perceived wellbeing.

Self-rated mental health was assessed by the single item measures of self-rated mental distress, based on a five-point scale re-coded from the following responses: excellent, very Good, good, fair, or poor (Ahmad et al., 2014).

Perceived wellbeing was assessed the Hong Kong version of Personal Wellbeing Index (PWI-7) (Lau et al., 2005). PWI-7 is a 7-item indicator to evaluate subjective wellbeing of an individual, which covers the following dimensions: self-rated health (personal health), standard of living, achievements in life, feeling safe (personal safety), the groups of people you belong to (community-connectedness), security for the future (future security), and interpersonal relationships. For each item, participants would rate between 0 and 10. “0” indicated no satisfaction at all and “10” indicated completely satisfied.

Factors regarding health-related lifestyle were (1) smoking status and (2) alcohol consumption. This study assessed alcohol consumption of each participant based on the following groups: never drink, monthly drinkers, weekly drinkers.

Socio-demographic characteristics included (1) gender, (2) age, (3) education level, (4) monthly household income, and (5) living with persons with special needs (e.g., with disability or child). Age was sub-grouped as follows: 18–29, 30–39, 40–49, 50–59, 60–69, and >=70. Educational level was sub-grouped as follows: sub-degree or above, secondary school and below. Monthly household income was sub-grouped as follows: > = HKD$20,000 and <HKD$20000.

### Statistical analysis

This study applied a two-stage approach to evaluate the shifts in associations between individual risk factors and perceived pain intensity. A subgroup analysis based on paired samples t-test was first conducted to evaluate the difference in perceived pain intensity among various participants before and after pandemic. The results of paired samples t-test for each subgroup were compared with the results from all participants separately, which the group of all participants was defined as the baseline group in this study. For the subgroups (1) with >0.05 average score higher perceived pain intensity than the baseline group and (2) the difference in perceived pain intensity before and after pandemic was significant (*p*-value <0.05), these were defined as individuals with severe increase in perceived pain intensity. For the subgroups also with 95% lower confidence interval (CI) and upper CI higher than corresponding values from the baseline group, we defined these as subgroups with the most significant increase in perceived pain intensity compared to the other individuals.

Based on the risk factors with an average NRS of perceived pain intensity 0.05 higher than the baseline group, we selected these factors as major variables to evaluate the effects on perceived pain intensity before and after pandemic. Three multivariate regressions were applied in this study: (1) the effects from pre-pandemic conditions of individual risk factors on pre-pandemic perceived pain intensity, (2) the effects from pre-pandemic conditions of individual risk factors on post-pandemic perceived pain intensity, and (3) the effects from post-pandemic conditions of individual risk factors on post-pandemic perceived pain intensity. The three sets of models were to include both long-term and short-term risk effects in the analysis. Beta (ß) coefficients and the 95% CIs were reported in this analysis.

All analyses were performed by IBM SPSS.

## Results

### Vulnerable subgroups

Based on 3,237 participants aged ≥15 with valid samples, 15.51% (*n* = 502) reported chronic pain. The average NRS in pre-pandemic and post-pandemic was 1.62 and 2.17, respectively (Table 1). Additionally, about 20.95% (*n* = 678) and 30.58% (*n* = 990) participants were with NRS > = 4 for their pre-pandemic and post-pandemic conditions. These indicated an increase in perceived pain intensity.

**Table 1:** Data summary—NRS of pre-pandemic and post- pandemic perceived pain intensity among vulnerable subgroups and all participants.

			Mean	*N*	Std.	Std. error mean
Baseline: all participants		Pain score (post-pandemic)	2.17	3,237	2.71	0.05
		Pain score (pre-pandemic)	1.62	3,237	2.28	0.04
Lifestyle	**Smoked**	**Pain score (post-pandemic)**	**2.43**	**259**	**2.79**	**0.17**
		**Pain score (pre-pandemic)**	**1.76**	**259**	**2.31**	**0.14**
	**Never drink**	**Pain score (post-pandemic)**	**2.23**	**1777**	**2.71**	**0.06**
		**Pain score (pre-pandemic)**	**1.67**	**1777**	**2.30**	**0.05**
	Monthly drinkers	Pain score (post-pandemic)	2.14	1,153	2.74	0.08
		Pain score (pre-pandemic)	1.62	1,153	2.30	0.07
	Weekly drinkers	Pain score (post-pandemic)	1.88	281	2.55	0.15
		Pain score (pre-pandemic)	1.33	281	1.99	0.12
Medical history	**Insomnia**	**Pain score (post-pandemic)**	**3.24**	**524**	**2.89**	**0.13**
		**Pain score (pre-pandemic)**	**2.58**	**524**	**2.64**	**0.12**
	**Lung**	**Pain score (post-pandemic)**	**3.58**	**52**	**2.93**	**0.41**
		**Pain score (pre-pandemic)**	**2.29**	**52**	**2.40**	**0.33**
	**Cardio**	**Pain score (post-pandemic)**	**2.73**	**122**	**2.67**	**0.24**
		**Pain score (pre-pandemic)**	**2.11**	**122**	**2.25**	**0.20**
	**Mental**	**Pain score (post-pandemic)**	**4.08**	**96**	**3.05**	**0.31**
		**Pain score (pre-pandemic)**	**3.01**	**96**	**2.80**	**0.29**
	**Cancer**	**Pain score (post-pandemic)**	**3.04**	**52**	**2.72**	**0.38**
		**Pain score (pre-pandemic)**	**2.42**	**52**	**2.37**	**0.33**
Personal wellbeing and psychological distress	Self-rated mental health: good	Pain score (post-pandemic)	2.03	2,330	2.62	0.05
		Pain score (pre-pandemic)	1.51	2,330	2.19	0.05
	**Self-rated mental health: bad**	**Pain score (post-pandemic)**	**2.53**	**871**	**2.88**	**0.10**
		**pain score (pre-pandemic)**	**1.95**	**871**	**2.48**	**0.08**
	Standard of living: good	Pain score (post-pandemic)	2.09	2,593	2.66	0.05
		Pain score (pre-pandemic)	1.53	2,593	2.21	0.04
	**Standard of living: bad**	**Pain score (post-pandemic)**	**2.48**	**644**	**2.87**	**0.11**
		**Pain score (pre-pandemic)**	**2.00**	**644**	**2.51**	**0.10**
	Personal Health: good	Pain score (post-pandemic)	2.07	2,736	2.66	0.05
		Pain score (pre-pandemic)	1.51	2,736	2.20	0.04
	**Personal Health: bad**	**Pain score (post-pandemic)**	**2.71**	**500**	**2.90**	**0.13**
		**Pain score (pre-pandemic)**	**2.25**	**500**	**2.58**	**0.12**
	Achievements in Life: good	Pain score (post-pandemic)	2.07	2,165	2.69	0.06
		Pain score (pre-pandemic)	1.50	2,165	2.21	0.05
	**Achievements in Life: bad**	**Pain score (post-pandemic)**	**2.36**	**1,063**	**2.74**	**0.08**
		**Pain score (pre-pandemic)**	**1.86**	**1,063**	**2.39**	**0.07**
	Interpersonal Relationships: good	Pain score (post-pandemic)	2.10	2,686	2.69	0.05
		Pain score (pre-pandemic)	1.55	2,686	2.23	0.04
	**Interpersonal Relationships: bad**	**Pain score (post-pandemic)**	**2.49**	**549**	**2.79**	**0.12**
		**Pain score (pre-pandemic)**	**2.00**	**549**	**2.48**	**0.11**
	Personal Safety: good	Pain score (post-pandemic)	2.09	2,477	2.67	0.05
		Pain score (pre-pandemic)	1.54	2,477	2.23	0.04
	**Personal Safety: bad**	**Pain score (post-pandemic)**	**2.42**	**755**	**2.81**	**0.10**
		**Pain score (pre-pandemic)**	**1.89**	**755**	**2.42**	**0.09**
	Community-Connectedness: good	Pain score (post-pandemic)	2.13	2,229	2.69	0.06
		Pain score (pre-pandemic)	1.55	2,229	2.23	0.05
	**Community-Connectedness: bad**	**Pain score (post-pandemic)**	**2.23**	**1,003**	**2.74**	**0.09**
		**Pain score (pre-pandemic)**	**1.79**	**1,003**	**2.38**	**0.08**
	Future Security: good	Pain score (post-pandemic)	2.11	2,229	2.69	0.06
		Pain score (pre-pandemic)	1.54	2,229	2.21	0.05
	**Future Security: bad**	**Pain score (post-pandemic)**	**2.29**	**1,001**	**2.76**	**0.09**
		**Pain score (pre-pandemic)**	**1.82**	**1,001**	**2.42**	**0.08**
Socio-demographic characteristics	Gender: Male	Pain score (post-pandemic)	1.87	1775	2.52	0.06
		Pain score (pre-pandemic)	1.42	1775	2.10	0.05
	**Gender: Females**	**Pain score (post-pandemic)**	**2.53**	**1,403**	**2.89**	**0.08**
		**Pain score (pre-pandemic)**	**1.88**	**1,403**	**2.46**	**0.07**
	Age: 18–29	Pain score (post-pandemic)	1.67	451	2.54	0.12
		Pain score (pre-pandemic)	1.30	451	2.18	0.10
	Age: 30–39	Pain score (post-pandemic)	2.14	803	2.74	0.10
		Pain score (pre-pandemic)	1.53	803	2.24	0.08
	Age: 40–49	Pain score (post-pandemic)	2.17	775	2.75	0.10
		Pain score (pre-pandemic)	1.62	775	2.30	0.08
	**Age: 50–59**	**Pain score (post-pandemic)**	**2.34**	**661**	**2.73**	**0.11**
		**Pain score (pre-pandemic)**	**1.76**	**661**	**2.29**	**0.09**
	**Age: 60–69**	**Pain score (post-pandemic)**	**2.49**	**453**	**2.70**	**0.13**
		**Pain score (pre-pandemic)**	**1.95**	**453**	**2.35**	**0.11**
	Age: > 70	Pain score (post-pandemic)	2.14	79	2.58	0.29
		Pain score (pre-pandemic)	1.59	79	2.17	0.24
	**Education: Secondary school or below**	**Pain score (post-pandemic)**	**2.57**	**551**	**2.89**	**0.12**
		**Pain score (pre-pandemic)**	**1.87**	**551**	**2.36**	**0.10**
	Education: sub-degree or above	Pain score (post-pandemic)	2.09	2,674	2.67	0.05
		Pain score (pre-pandemic)	1.58	2,674	2.26	0.04
	**Household income: < HKD$ 20,000**	**Pain score (post-pandemic)**	**2.64**	**432**	**2.86**	**0.14**
		**Pain score (pre-pandemic)**	**2.00**	**432**	**2.46**	**0.12**
	Household income: > = HKD$ 20,000	Pain score (post-pandemic)	2.09	2,762	2.68	0.05
		Pain score (pre-pandemic)	1.57	2,762	2.25	0.04
	**Living with persons with special needs**	**Pain score (post-pandemic)**	**2.52**	**405**	**2.87**	**0.14**
		**Pain score (pre-pandemic)**	**1.74**	**405**	**2.32**	**0.12**
	Not living with persons with special needs	Pain score (post-pandemic)	2.11	2,755	2.67	0.05
		Pain score (pre-pandemic)	1.61	2,755	2.27	0.04

Vulnerable subgroups were categorized based on self-reported (pre-pandemic) socio-demographic information. Bold text indicated vulnerable subgroup with an average NRS higher than baseline (all participants).

For those with medical history, their average NRS in both pre- and post-pandemic were generally higher than NRS from all participants. Participants with known psychiatric diseases had the highest average NRS (average NRS: 3.01 in pre-pandemic, 4.08 in post-pandemic), seconded by those with respiratory diseases (average NRS: 2.29 in pre-pandemic, 3.58 in post-pandemic) and insomnia (average NRS: 2.58 in pre-pandemic, 3.24 in post-pandemic). Participants with cardiovascular diseases and cancer also reported a higher average NRS in both pre- and post-pandemic period compared to scores for all participants.

Individuals with low personal wellbeing and high psychological distress were also vulnerable subgroups of pain in both pre- and post-pandemic period. Those who self-rated to have poor personal health were with the highest average score of perceived pain intensity (average NRS: 2.25 in pre-pandemic, 2.71 in post-pandemic), seconded by those with low self-rated mental health (average NRS: 1.95 in pre-pandemic, 2.53 in post-pandemic) and poor personal relationship (average NRS: 2.00 in pre-pandemic, 2.49 in post-pandemic). Participants with lower scores of standards of living, life achievement, personal safety, community-connectedness, and future security also had higher average NRS in both pre- and post-pandemic period compared to the baseline group.

For lifestyle, participants who smoked were with higher average NRS in both pre- and post-pandemic period (average NRS: 1.76 in pre-pandemic, 2.43 in post-pandemic). Surprisingly, those who declared to never drink any alcohol beverages had higher average NRS than all participants but not those who were weekly or monthly drinkers.

Among all factors related to socio-demographic characteristics, those who were females, lower education (secondary school or below), low household income (<HKD$ 20,000), lived with persons with special needs and aged between 50 and 69 were individuals with higher average NRS. The subgroup with the highest average NRS was low household income (average NRS: 2.00 in pre-pandemic, 2.64 in post-pandemic), seconded by lower education (average NRS: 1.87 in pre-pandemic, 2.57 in post-pandemic), females (average NRS: 1.88 in pre-pandemic, 2.53 in post-pandemic), and lived with persons with special needs (average NRS: 1.74 in pre-pandemic, 2.52 in post-pandemic).

### Shifts in perceived pain intensity

Overall, participants have experienced a slightly higher perceived pain intensity after pandemic. It was a 0.54 [CI: 0.49,0.59] increase in NRS among all participants (Table 2).

**Table 2:** Increase in perceived pain intensity after pandemic.

		Paired differences					*t*	df	*p*-value
		Mean	Std	Std. error mean	95% CI				
					Lower	Upper			
Baseline: all participants		0.54	1.44	0.03	0.49	0.59	21.374	3,236	0.000
Medical history	**Insomnia**	**0.66**	**1.56**	**0.07**	**0.53**	**0.80**	**9.693**	**523**	**0.000**
	**Respiratory diseases**	**1.29**	**2.30**	**0.32**	**0.65**	**1.93**	**4.048**	**51**	**0.000**
	**Cardiovascular diseases**	**0.61**	**1.55**	**0.14**	**0.34**	**0.89**	**4.394**	**121**	**0.000**
	**Known psychiatric diseases**	**1.07**	**2.17**	**0.22**	**0.63**	**1.51**	**4.838**	**95**	**0.000**
	**Cancer**	**0.62**	**1.46**	**0.20**	**0.21**	**1.02**	**3.045**	**51**	**0.004**
Personal wellbeing and psychological distress	Self-rated mental health: good	0.51	1.40	0.03	0.46	0.57	17.698	2,329	0.000
	Self-rated mental health: bad	0.57	1.48	0.05	0.47	0.67	11.438	870	0.000
	standard of living: good	0.56	1.46	0.03	0.50	0.61	19.476	2,592	0.000
	standard of living: bad	0.48	1.38	0.05	0.37	0.59	8.815	643	0.000
	Personal Health: good	0.56	1.45	0.03	0.50	0.61	20.098	2,735	0.000
	Personal Health: bad	0.46	1.40	0.06	0.34	0.58	7.339	499	0.000
	Achievements in Life: good	0.57	1.48	0.03	0.50	0.63	17.770	2,164	0.000
	Achievements in Life: bad	0.50	1.37	0.04	0.41	0.58	11.808	1,062	0.000
	Interpersonal Relationships: good	0.55	1.45	0.03	0.50	0.61	19.789	2,685	0.000
	Interpersonal Relationships: bad	0.49	1.41	0.06	0.37	0.61	8.098	548	0.000
	Personal Safety: good	0.55	1.44	0.03	0.49	0.60	18.825	2,476	0.000
	Personal Safety: bad	0.54	1.46	0.05	0.43	0.64	10.081	754	0.000
	Community-Connectedness: good	0.59	1.49	0.03	0.53	0.65	18.631	2,228	0.000
	Community-Connectedness: bad	0.44	1.34	0.04	0.36	0.52	10.433	1,002	0.000
	Future Security: good	0.57	1.47	0.03	0.51	0.63	18.330	2,228	0.000
	Future Security: bad	0.48	1.38	0.04	0.39	0.56	10.933	1,000	0.000
Lifestyle	**Smoked**	**0.68**	**1.59**	**0.10**	**0.48**	**0.87**	**6.826**	**258**	**0.000**
	Never drink	0.55	1.48	0.04	0.48	0.62	15.770	1776	0.000
	Monthly drinkers	0.52	1.41	0.04	0.44	0.60	12.575	1,152	0.000
	Weekly drinkers	0.55	1.36	0.08	0.39	0.71	6.744	280	0.000
Socio-demographic characteristics	Gender: Males	0.45	1.25	0.03	0.39	0.51	15.170	1774	0.000
	**Gender: Females**	**0.65**	**1.64**	**0.04**	**0.57**	**0.74**	**14.943**	**1,402**	**0.000**
	Age: 18–29	0.37	1.16	0.05	0.26	0.48	6.781	450	0.000
	**Age: 30–39**	**0.60**	**1.44**	**0.05**	**0.50**	**0.70**	**11.861**	**802**	**0.000**
	Age; 40–49	0.55	1.57	0.06	0.44	0.66	9.790	774	0.000
	Age: 50–59	0.58	1.48	0.06	0.47	0.70	10.114	660	0.000
	Age: 60–69	0.53	1.46	0.07	0.40	0.67	7.775	452	0.000
	Age: > 70	0.54	1.29	0.15	0.26	0.83	3.753	78	0.000
	**Education: Secondary school or below**	**0.70**	**1.70**	**0.07**	**0.55**	**0.84**	**9.626**	**550**	**0.000**
	Education: sub-degree or above	0.51	1.39	0.03	0.46	0.56	19.099	2,673	0.000
	**Household income: < HKD$ 20,000**	**0.63**	**1.66**	**0.08**	**0.48**	**0.79**	**7.933**	**431**	**0.000**
	Household income: > = HKD$ 20,000	0.52	1.41	0.03	0.47	0.58	19.584	2,761	0.000
	**living with persons with special needs**	**0.78**	**1.73**	**0.09**	**0.61**	**0.95**	**9.067**	**404**	**0.000**
	not living with persons with special needs	0.50	1.38	0.03	0.45	0.55	19.147	2,754	0.000

Bold text indicated significant vulnerable subgroup with an increase in average NRS > 0.05 than the baseline (all participants). Text highlighted in read indicated the subgroup with the most significant increase in perceived pain intensity (average NRS and 95% CI higher than the baseline).

There were 11 subgroups with severe increase in perceived pain intensity after pandemic: individual who were (1) smoked, (2) with insomnia, (3) with respiratory diseases, (4) with cardiovascular diseases, (5) with known psychiatric diseases, (6) with cancer, (7) female, (8) age between 30 and 39, (9) lower education (secondary school or below), (10) low household income (<HKD$ 20,000), and (11) lived with persons with special needs. Based on the difference in average and 95% CIs, subgroups with respiratory disease, known psychiatric diseases and those living with persons with special needs were the individuals experienced with the most significant increase in perceived pain intensity after pandemic. Individuals with respiratory disease had a 1.29 [CI:0.65, 1.93] increase in NRS, individuals with known psychiatric diseases had a 1.07 [CI: 0.63, 1.51] increase in NRS, and individuals living with persons with special needs had a 0.78 [CI:0.61, 0.95] increase in NRS.

### Effects of risk factors

For pre-pandemic conditions (Table 3), the major individual risk factors affecting perceived pain intensity were as follows: (1) insomnia, (2) known psychiatric diseases, (3) females, and (4) low household income. Specifically, individuals who were females, low household income (<HKD$20,000), with insomnia, and with known psychiatric diseases had 0.395 [CI: 0.238, 0.551], 0.237 [CI: 0.006, 0469], 0.493 [CI: 0.386, 0.600], and 0.169 [CI: 0.076, 0.262] higher NRS than the comparison groups.

**Table 3:** The effects from pre-pandemic conditions of individual risk factors on pre-pandemic perceived pain intensity.

Risk factors	Estimate	SE	*t*-value	95%CI		*P*-value
				Lower	Upper	
Smoked (Pre)	−0.004	0.147	−0.027	−0.292	0.284	0.979
**Insomnia (Pre)**	**0.493**	**0.055**	**9.040**	**0.386**	**0.600**	**0.000**
Respiratory diseases (Pre)	0.046	0.105	0.437	−0.160	0.252	0.662
Cardiovascular diseases (Pre)	0.085	0.052	1.634	−0.017	0.187	0.102
**Known psychiatric diseases (Pre)**	**0.169**	**0.047**	**3.573**	**0.076**	**0.262**	**0.000**
Cancer (Pre)	0.083	0.052	1.586	−0.020	0.185	0.113
**Female**	**0.395**	**0.080**	**4.953**	**0.238**	**0.551**	**0.000**
Age: 30–39	−0.013	0.023	−0.545	−0.058	0.033	0.586
Education: Secondary school or below	0.181	0.108	1.676	−0.031	0.394	0.094
**Household income: <HKD$ 20,000**	**0.237**	**0.118**	**2.008**	**0.006**	**0.469**	**0.045**
Living with persons with special needs (Pre)	0.069	0.118	0.580	−0.163	0.301	0.562

Those with insomnia and known psychiatric diseases before pandemic as well as females remained as subgroups with higher perceived pain intensity after pandemic (Table 4). The effect sizes of these individual risk factors were higher than the effect sizes affect perceived pain intensity in pre-pandemic. Specifically, individuals with insomnia before pandemic were the most significant group, with a 0.519 [CI: 0.392, 0.646] higher NRS compared with the control group. Furthermore, having respiratory disease and living with persons with special needs during pre-pandemic as well as low education (secondary school or below) were additional risk factors.

**Table 4:** The effects from pre-pandemic conditions of individual risk factors on post-pandemic perceived pain intensity.

Risk factors	Estimate	SE	*t*-value	95%CI		*P*-value
				Lower	Upper	
Smoked (Pre)	0.107	0.174	0.615	−0.234	0.448	0.539
**Insomnia (Pre)**	**0.519**	**0.065**	**8.033**	**0.392**	**0.646**	**0.000**
**Respiratory diseases (Pre)**	**0.267**	**0.125**	**2.141**	**0.022**	**0.511**	**0.032**
Cardiovascular diseases (Pre)	0.096	0.062	1.552	−0.025	0.217	0.121
**Known psychiatric diseases (Pre)**	**0.250**	**0.056**	**4.450**	**0.140**	**0.360**	**0.000**
Cancer (Pre)	0.074	0.062	1.202	−0.047	0.196	0.229
**Female**	**0.582**	**0.094**	**6.166**	**0.397**	**0.767**	**0.000**
Age: 30–39	0.014	0.027	0.504	−0.040	0.067	0.614
**Education: Secondary school or below**	**0.351**	**0.128**	**2.733**	**0.099**	**0.602**	**0.006**
Household income: <HKD$ 20,000	0.265	0.140	1.896	−0.009	0.540	0.058
**Living with persons with special needs (Pre)**	**0.321**	**0.140**	**2.287**	**0.046**	**0.596**	**0.022**

Considering the effects from post-pandemic conditions of individual risk factors on post-pandemic perceived pain intensity (Table 5), (1) insomnia, (2) known psychiatric diseases, (3) respiratory disease, (4) cardiovascular disease, (5) females, (6) low household income, and (7) low education (secondary school or below) were significant risk factors. Effect sizes of insomnia, respiratory disease, and known psychiatric disease were much higher than the effect sizes in pre-pandemic. Individuals with insomnia were the most significant group, which had 1.018 [CI: 0.814, 1.221] higher NRS than the comparison groups after pandemic.

**Table 5:** The effects from post-pandemic conditions of individual risk factors on post-pandemic perceived pain intensity.

Risk factors	Estimate	SE	*t*-value	95%CI		*P*-value
				Lower	Upper	
Smoked (Post)	0.038	0.171	0.219	−0.298	0.373	0.826
**Insomnia (Post)**	**1.018**	**0.104**	**9.784**	**0.814**	**1.221**	**0.000**
**Respiratory diseases (Post)**	**0.856**	**0.312**	**2.744**	**0.245**	**1.468**	**0.006**
**Cardiovascular diseases (Post)**	**0.559**	**0.214**	**2.615**	**0.140**	**0.978**	**0.009**
**Known psychiatric diseases (Post)**	**0.951**	**0.208**	**4.571**	**0.543**	**1.358**	**0.000**
Cancer (Post)	0.693	0.362	1.914	−0.017	1.402	0.056
**Female**	**0.557**	**0.094**	**5.957**	**0.374**	**0.740**	**0.000**
Age: 30–39	0.056	0.109	0.512	−0.157	0.269	0.609
**Education: Secondary school or below**	**0.335**	**0.127**	**2.642**	**0.086**	**0.583**	**0.008**
**Household income: <HKD$ 20,000**	**0.284**	**0.138**	**2.060**	**0.014**	**0.555**	**0.039**
Living with persons with special needs (Post)	0.192	0.134	1.435	−0.070	0.455	0.151

## Discussion

We investigated the shifts in risk factors of perceived pain intensity after pandemic among 3,237 urban individuals in Hong Kong. Those with medical history (respiratory diseases, cardiovascular diseases, cancer, known psychiatric diseases, insomnia) and personal wellbeing (personal health, standard of living, achievements in life, personal safety, community-connectedness, future security, and interpersonal relationships) and self-rated mental health were continuously with high perceived pain intensity in pre- and post-pandemic. Smoked and socio-demographic deprived individuals (female, older adults, low education, low income, living with special needs) were also with higher perceived pain intensity. These results were consistent with previous studies; for example, females and individuals who smoked were usually more sensitive to pain and would rate a higher perceived pain intensity based on NRS (Levine and De Simone, 1991; Robinson et al., 2001; Weingarten et al., 2008). Chronic pain was often observed among cancer survivors (Burton et al., 2007; Levy et al., 2008). Low education and low income can affect pain-related healthy behaviors and pain treatment of oneself (Atkins and Mukhida, 2022). Older adults were usually frail, resulting in a higher risk of chronic pain (Reyes et al., 2019).

Advanced from previous studies, our results indicated the subgroups with greater increase in perceived pain intensity since pandemic. The results were interesting, as not all vulnerable subgroups had severer increases in perceived pain intensity than the general populations. Although individuals with low personal wellbeing and mental distress were vulnerable subgroups, the increase of perceived pain intensity among these individuals since pandemic was not as high as the baseline group. In contrast, those with known psychiatric diseases were with significant increase in perceived pain intensity after pandemic. It is important to note that average NRS among individuals with known psychiatric diseases was >4.0 after pandemic. This result is critical, as previous studies have widely adopted NRS > =4 as cutoff of moderate and severe pain (Lahtinen et al., 2006; Ledowski et al., 2006; Gerbershagen et al., 2011). As chronic pain is a pain experience persisting for at least 3 months (Cheung et al., 2017; Merskey, 1986; Ng et al., 2002) and those who experiences pain throughout the pandemic should have experience pain much longer than this, individuals with known psychiatric diseases who also had NRS > =4 were in urgent need of pain treatment.

The difference between individuals with low personal wellbeing / mental distress and known psychiatric diseases/insomnia could be explained by medical assumptions. Personal wellbeing and mental distress are emotional responses. Although negative emotions can be lasted long and some studies suggested that adverse psychological conditions during pandemic could induce post-traumatic stress disorder’s symptoms (Liu et al., 2020), these negative emotional responses could be reduced once the individuals built the psychological resilience to cope with adverse situations. As such, the impacts of personal wellbeing and mental distress could be varied in a short-term (Simons et al., 2014) and may be unable to result in significant side effects, such as increase in perceived level of pain. However, having psychiatric diseases may not only be an emotional/psychological issue, but also a health condition that continuously affects neurological and physiological responses of an individual (Lautenbacher and Krieg, 1994). Insomnia is also a health condition that is related to autonomic nervous system (McCall et al., 2023) and neurological disorders (Mayer et al., 2011). Previous studies have found the associations between psychiatric disorders/insomnia and perceived pain intensity (Velly and Mohit, 2018; Wei et al., 2018). The change in pain perception can be due to a pathophysiological mechanism controlled by neurochemical and neurohormonal functions, which is usually affected by the processes of psychiatric diseases (Lautenbacher and Krieg, 1994). As perception of pain is highly related to nervous system (Hudson, 2000), the impacts from known psychiatric diseases and insomnia can be severe and in a long-term.

The severe increase in perceived pain intensity among individuals with cardiovascular and respiratory diseases can be due to the associations between cardiovascular and respiratory functions and post-covid symptoms (long COVID). Previous studies have found that COVID infection can affect cardiovascular system in an individual, which can result in myocardial injury, myocarditis, acute myocardial infarction, heart failure, dysrhythmias, and venous thromboembolic events (Long et al., 2020). The weakened cardiovascular and respiratory systems after COVID infection may the issue to develop a post-covid symptom of pain among individuals. This can partially be explained by our results of multivariate regressions. Specifically, cardiovascular and respiratory diseases were insignificant for the pre-pandemic model; however, these become significant risk factors for the post-pandemic models.

The results regarding socio-demographic effects were interesting. Older ages (50–69) showed higher perceived pain intensity in both pre- and post-pandemic period in compared with the general populations; however, the degree in pain increase was not as high as the baseline group. Surprisingly, individuals aged between 30 and 39 had a severe increase in perceived pain intensity. Although the pathological mechanism was unclear, it might be due to the change in lifestyles and behaviors among working-aged adults. Further studies should be conducted to investigate the underlying cause of pain increase among the working-aged adults. Due to emotional and physical burdens of caregivers (Jones et al., 2011), living with special needs can be a cause of severe pain. As the pandemic has induced social restrictions and affected willingness of taking formal medical treatment in Hong Kong, burdens of family caregivers were increased. It may be the reason why living with special needs became one of the vulnerable subgroups with the greater increase in perceived pain intensity since pandemic.

The above results highlighted the needs to develop up-to-date interventions targeting vulnerable populations, particularly individuals with known psychiatric diseases and insomnia, for pain reduction. This is critical in pain management, as traditional clinical practice usually suggested pain as a physiological response. Therefore, common methods to reduce pain intensity were mainly based on various types of drugs. However, as suggested by the biopsychosocial model of pain (Turk et al., 2011), perceived pain intensity can also be a psychological outcome. Therefore, only taking pain relievers may not be always helpful. More importantly, there are already serious issues regarding opioid overdose worldwide (e.g., “opioid epidemic” in the United States) and this opioid crisis is being severer after COVID-19 (Manchikanti et al., 2012; Laing and Donnelly, 2024; Blair et al., 2023; Simha et al., 2023). The unfortunate side of this opioid crisis was the significant association with psychiatric diseases and insomnia (Sampasa-Kanyinga et al., 2024; Chamoun et al., 2023). Therefore, it is important to develop specific programs for mental support so that pain clinicians and related professionals can reduce perceived pain intensity among patients from both physiological and psychological perspectives.

Several limitations should be noted. It was a population-based study conducted based on an existing online panel. Those with difficulties using internet may be unable to conduct the survey. Future studies could consider combining online and face-to-face surveys for enhancing the analysis. Pre-pandemic records were assessed based on retrospective information self-reported from the participants. It may be due to a subjective bias. A follow-up study should be conducted to develop a comprehensive longitudinal dataset for analysis. Furthermore, some risk factors causing pain may be interlocked. Structural equation modelling should be conducted in the future to analyze the mechanisms causing pain via different pathways. Finally, due to the nature of online-based survey, specific pain-related factors, such as pain catastrophizing and kinesiophobia (Landmark et al., 2024; Haythornthwaite et al., 2024), were not able to assess. Future studies should consider using a mixed-methods study with qualitative and quantitative data to improve the assessment.

## Conclusion

Having known psychiatric diseases and insomnia were the most vulnerable subgroups of pain in Hong Kong. Individuals with psychiatric diseases were not only with higher perceived pain intensity in both pre- and post-pandemic period in compared with general population, but also a severe increase in perceived pain intensity after pandemic, as well as continuously being significant risk factors causing pain. As psychiatric diseases and insomnia can be health conditions related to neurological and physiological responses, how to develop strategies for enhancing neurological and physiological functions of oneself for pain management and reduction of post-COVID symptoms is critical.

## Data Availability

The original contributions presented in the study are included in the article/supplementary material, further inquiries can be directed to the corresponding author/s.
